# EBV-gp350 Confers B-Cell Tropism to Tailored Exosomes and Is a Neo-Antigen in Normal and Malignant B Cells—A New Option for the Treatment of B-CLL

**DOI:** 10.1371/journal.pone.0025294

**Published:** 2011-10-10

**Authors:** Romana Ruiss, Simon Jochum, Ralph Mocikat, Wolfgang Hammerschmidt, Reinhard Zeidler

**Affiliations:** 1 Department of Gene Vectors, Helmholtz-Zentrum, Munich, Germany; 2 Institut für Molekulare Immunologie, Helmholtz-Zentrum, Munich, Germany; 3 Department of Otorhinolaryngology, Ludwig-Maximilians-Universität, Munich, Germany; Technische Universitaet Muenchen, Germany

## Abstract

gp350, the major envelope protein of Epstein-Barr-Virus, confers B-cell tropism to the virus by interacting with the B lineage marker CD21. Here we utilize gp350 to generate tailored exosomes with an identical tropism. These exosomes can be used for the targeted co-transfer of functional proteins to normal and malignant human B cells. We demonstrate here the co-transfer of functional CD154 protein on tailored gp350+ exosomes to malignant B blasts from patients with B chronic lymphocytic leukemia (B-CLL), rendering B blasts immunogenic to tumor-reactive autologous T cells. Intriguingly, engulfment of gp350+ exosomes by B-CLL cells and presentation of gp350-derived peptides also re-stimulated EBV-specific T cells and redirected the strong antiviral cellular immune response in patients to leukemic B cells. In essence, we show that gp350 alone confers B-cell tropism to exosomes and that these exosomes can be further engineered to simultaneously trigger virus- and tumor-specific immune responses. The simultaneous exploitation of gp350 as a tropism molecule for tailored exosomes and as a neo-antigen in malignant B cells provides a novel attractive strategy for immunotherapy of B-CLL and other B-cell malignancies.

## Introduction

Epstein-Barr virus (EBV) is an almost ubiquitous human gamma herpes virus that infects resting human B-lymphocytes, including B-CLL cells, with high efficacy [1], [2]. EBV's B-cell tropism is mainly due to gp350, the viral envelope glycoprotein that interacts with the cellular complement receptor 2 (CR2, CD21) [3] on B cells. In EBV seropositive individuals, gp350 mainly elicits CD4+ T-cell responses [4].

Exosomes are endosome-derived membrane vesicles, which are released by cells of diverse origin including dendritic cells, cancer cells [5] and EBV-infected B cells [6]. Exosomes bud from endosomal membranes and accumulate in multivesicular bodies, which eventually fuse with the cellular membrane and release the contained vesicles. Exosomes are rich in lipids and membrane proteins like MHC molecules, TNF-R and tetraspanins [5] but their specific composition depends on the cell of origin. Exosomes either fuse to the recipient cell membrane or are engulfed by phagocytic cells in such a way that exosome proteins are degraded and loaded onto MHC class II molecules [7]. Obviously, exosomes can deliver proteins as cargo in a very immunogenic manner so that they efficiently reactivate specific CD4+ T cell clones [8]. Hence, exosomes can induce strong and epitope-specific immune responses [9], [10] and can be used as an alternative to transfer strategies using gene vectors and as promising vaccines [11], [12].

Chronic lymphocytic leukemia of B-cell origin (B-CLL) is the most common adult leukemia in the Western hemisphere. B-CLL is considered as a prototypic disease undergoing immune evasion as the malignant cells lack important accessory and co-stimulatory molecules. Thus, despite their expression of high levels of surface MHC class I and II molecules, which presumably present tumor-associated antigenic epitopes, the leukemic cells tend to induce tumor-specific T-cell anergy. Typically, activated T cells from patients show a significantly reduced expression of CD40 ligand (CD154) or are completely CD154-negative [13]. As a consequence, T cells from B-CLL patients cannot activate cells through the CD40 receptor. This interaction, however, is essential for CD40 signaling and subsequent induction of other immune accessory molecules like CD80 and CD86, which increase the antigen-presenting capacity of normal and B-CLL cells. On the other hand, the EBV-specific cellular immunity is relatively intact in these patients [2]. To overcome the dysfunction of potentially tumor-reactive T cells from patients with B-CLL, several approaches have been developed relying on the stimulation of B-CLL cells through the CD40 pathway, including the ectopic expression of CD154 on the leukemic cells, and aiming at the self-stimulation of these cells [14]–[17]. In summary, immunotherapy of B-CLL is promising and CD154 is a potential candidate molecule to improve the patients' immune status and, eventually, the clinical outcome.

The robust cellular immunity in B-CLL patients against EBV [2] therefore prompted us to investigate the potential of tailored exosomes to redirect this immunity to malignant B cells. We present a novel approach for the targeted transfer of functional cellular proteins to B cells via tailored gp350+ exosomes. In this approach, gp350 has a dual function: (i) it confers B-cell tropism to exosomes so that they specifically co-transfer proteins of interest and (ii) it is a viral neo-antigen for these cells so that they efficiently reactive gp350-specific T cells. As a proof of concept, we show that tailored gp350+ exosomes can co-transfer functional CD154 as immune accessory molecule to B-CLL cells, which are subsequently stimulated to express surface molecules like CD54, CD80, CD86 and CD95 and stimulate autologous tumor- and EBV-specific T cells.

## Results

### EBV gp350 is packaged into exosomes, confers B-cell tropism, and reactivates specific T cells

**Figure 1:**
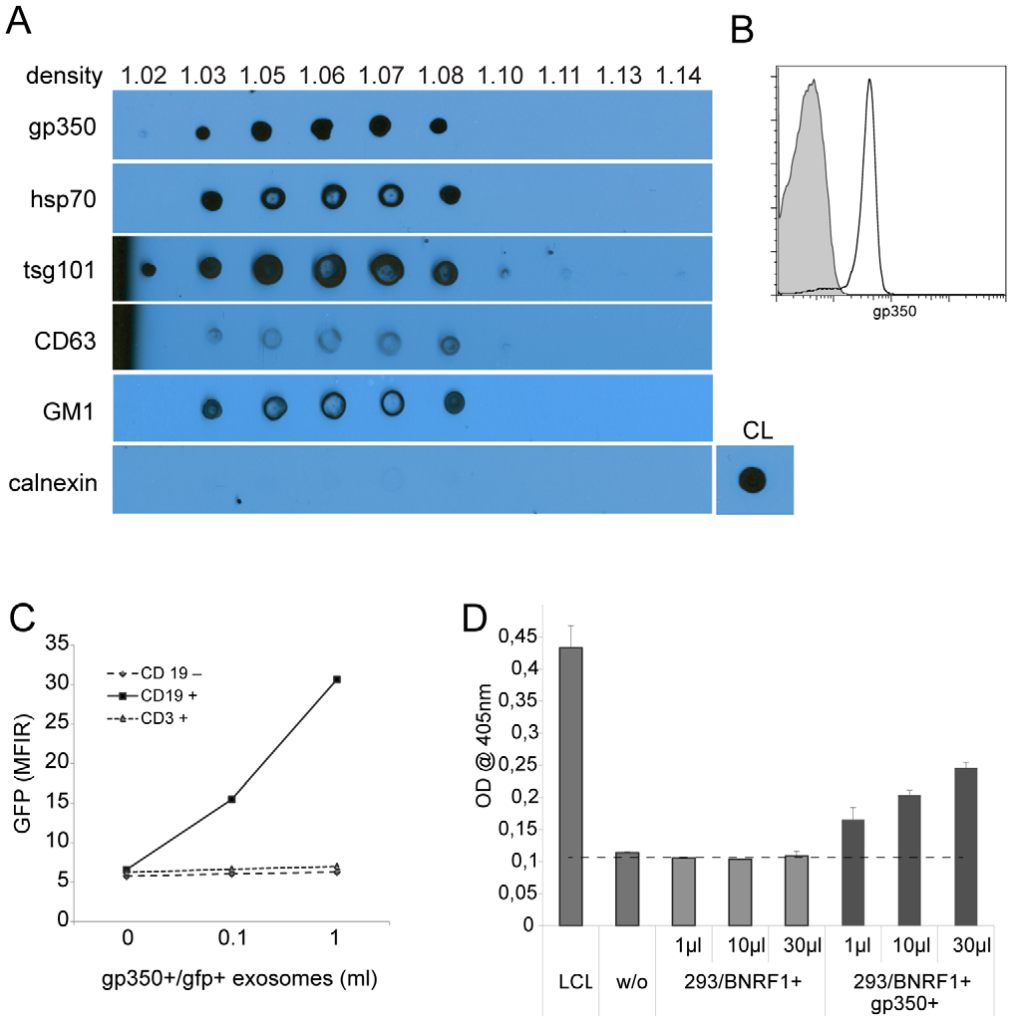
gp350 is incorporated into 293 exosomes and confers B cell tropism. (A) 293 cells were transfected with a gp350 expression plasmid, vesicles in the supernatant were purified as described and fractions of the OptiPrep™ gradients were spotted onto PVDF membrane. Immunoblots demonstrated that vesicles carry gp350 and that these vesicles co-sediment with vesicle that carry the exosome markers hsp70, tsg101, CD63 and ganglioside (GM)1 (www.exocarta.org). Calnexin is present in cell lysates (CL), its absence from exosomes demonstrated the purity of the preparation. (B) Latex beads were coated with gp350+ exosomes and stained with a gp350-specific antibody. gp350 is accessible and thus probably located in the exosome membrane (isotype control is shown as grey tinted histogram). (C) gp350 confers B-cell tropism to exosomes. PBMCs from a healthy donor were incubated for 18 h with gp350+/gfp+ exosomes and then analyzed for GFP fluorescence by flow cytometry. Only CD19+ B-cells stained positive, whereas an interaction of gp350+ exosomes with CD3+ T cells and CD19– cells (T and NK cells, monocytes) could not be observed. (D) B cells from a healthy donor were incubated with serial dilutions of exosomes from 293 cells transfected either with a BNRF1 expression plasmid or co-transfected with expression plasmids for BNRF1 (293/BNRF1) and gp350 (293/BNRF1+/gp350+) for one day. Then, autologous BNRF1-specific CD4+ T cells were added and their activation was measured with an IFN-γ ELISA one day later. T cells were activated at detectable levels by cells incubated with 293/BNRF1+/gp350+ exosomes but not with 293/BNRF1+ exosomes, indicating the relevance of gp350+ as a mediator of B cell tropism. An autologous EBV-infected lymphoblastoid cell line (LCL) was enclosed as a positive control.

EBV has a profound B-cell tropism that is mainly conveyed by gp350, which is the major EBV glycoprotein in the viral envelope and the ligand for cellular CD21 (CR2) on B cells. We knew from previous work that exosomes can transport ectopically expressed proteins such as *green fluorescent protein* (GFP), which is presumably present as a cargo in the exosomal lumen. In addition, several groups provided evidence that surface proteins are incorporated in exosome membranes [5]. We therefore asked whether gp350 could also become an integral part of exosomes and confer B-cell tropism to these vesicles. To answer this question, we co-transfected 293 cells with expression plasmids encoding *BLLF1*, the gene of gp350, and *gfp*. Three days later, we isolated vesicles from the supernatants of transfected HEK293 cells as described in Material and Methods and analyzed them by immunoblots for the presence of gp350 and exosome markers. Gp350 was detected in vesicles that floated at a density between 1.03 and 1.08 into an OptiPrep™ gradient, corresponding to a density between 1.13 to 1.18 in a sucrose gradient and thus in the density described for exosomes. The gradient also revealed the co-sedimentation of gp350 with the exosome markers hps70, tsg101 and CD63, indicating the nature of the gp350+ vesicles as exosomes (Figure 1A). Flow cytometry of exosomes coupled to latex beads revealed that gp350 is presumably located within the exosome membrane because it could be targeted with a specific antibody (Figure 1B). To demonstrate that gp350 confers B-cell tropism also to exosomes, we incubated gp350+/gfp+ exosomes with PBMCs from a healthy donor for one day and then quantified exosome binding by measuring GFP fluorescence by flow cytometry. This assay revealed that gp350+/gfp+ exosomes had an EBV-like tropism because they bound to CD19+ B cells but not to CD19-negative cells (Figure 1C).

Phagocytic cells engulf exosomes, process their proteins in lysosomes and present epitops in association with MHC class II molecules to CD4+ T cells [10]. To further utilize the potential of gp350+ exosomes to specifically transfer exogenous proteins to B cells, which, in turn, may activate specific T cells, we generated exosomes that carried BNRF1, the major tegument protein of EBV, either alone (BNRF1+) or together with gp350 (BNRF1+/gp350+). We then incubated purified CD19+ B cells with these exosomes overnight and used these PBMC as stimulators for an autologous BNRF1-specific CD4+ T-cell clone. As shown in Figure 1D, B cells incubated with BNRF1+/gp350+ exosomes activated the T-cell clone in a concentration-dependent manner whereas B cells incubated with BNRF1+ exosomes did not activate the T-cell clone. This result demonstrates the potential of gp350+ exosomes to transfer immunogenic foreign proteins to B cells that then can activate specific CD4+ T lymphocytes.

### Exosomes transfer functional CD154 to B-CLL cells

**Figure 2:**
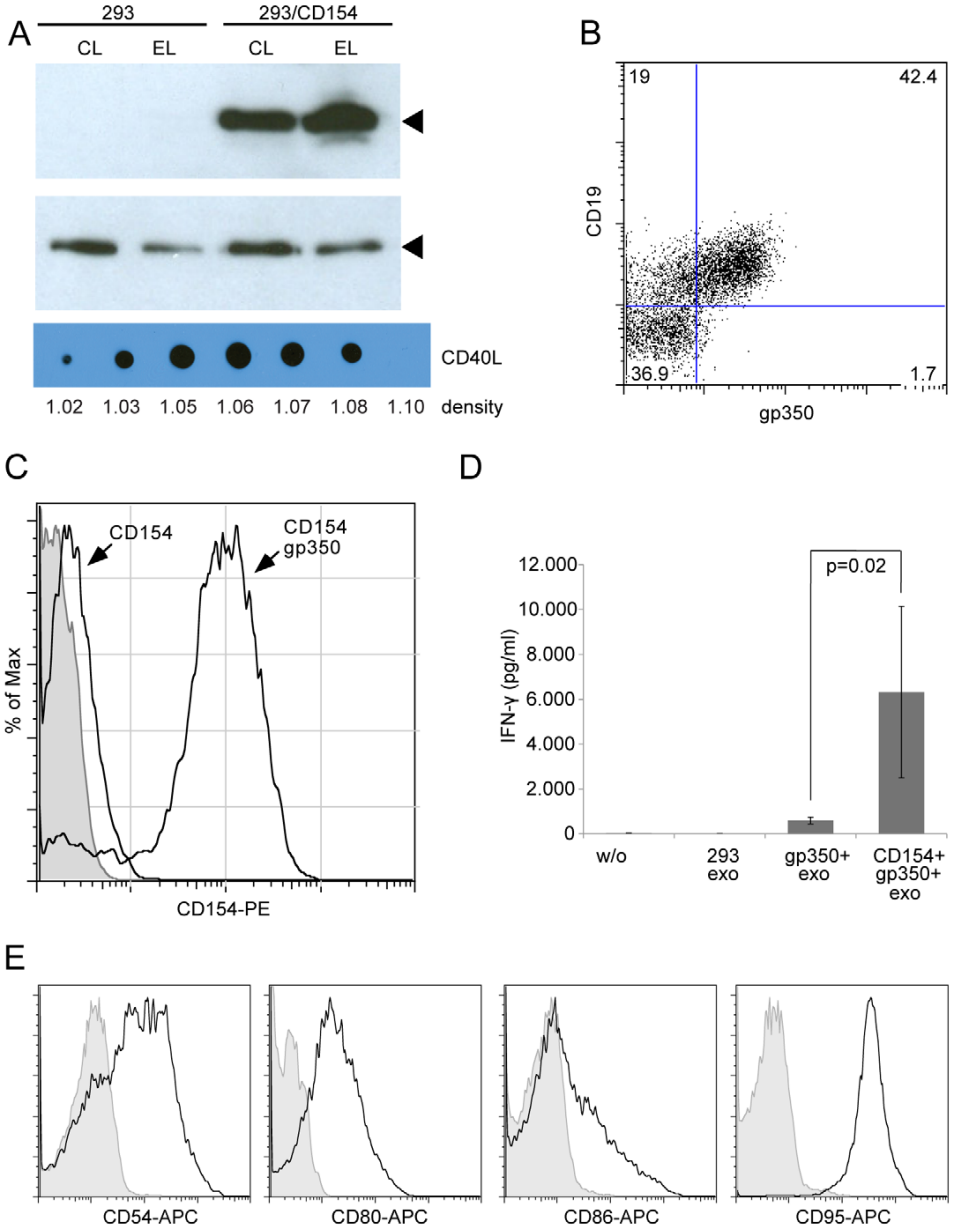
CD154 is packaged into exosomes as a functional protein and gp350 enhances the transfer of CD154 to B-CLL cells. (A) 293 cells were transiently transfected with an expression plasmid encoding CD154. An immunoblot with a CD154-specific antibody revealed that CD154 is highly expressed in transfected cell and that the protein is incorporated into exosomes. Shown are immunoblots of cell lysates (CL) and lysates from purified exosomes (EL) of normal and transfected 293 cells, incubated with CD154- (upper panel) and tsg101-specific antibodies (middle panel). An OptiPrep™ gradient revealed co-sedimentation of CD154- and gp350-carrying vesicles (see Figure 1A). (B) Exosomes carrying gp350+ specifically bind to CD19+ B cells from a B-CLL patient. Fresh B-CLL cells were incubated with purified exosomes from 293 cells transfected with a gp350 expression plasmid and binding of exosomes was measured by flow cytometry. (C) Exosomes carrying CD154 bind only weakly to B-CLL cells, probably through interaction with CD40, which is highly expressed on these cells. Binding of CD154-carrying exosomes is drastically enhanced by the co-expression of gp350. 293 cells were co-transfected with expression plasmids for CD154 and/or gp350. Fresh B-CLL cells were co-cultivated with CD154+/gp350– and CD154+/gp350+ exosomes for two days and binding of exosomes and thus transfer of CD154 was measured by flow cytometry. Filled histogram  =  CLL cells with exosomes from untransfected 293 cells. (D) Fresh HLA-DR13+ B-CLL cells were incubated with the exosomes indicated for one day and then co-incubated for another day with HLA-DR13-restriced gp350-specific CD4+ T cells. The transfer of functional CD154 renders B-CLL cells significantly more immunogenic (E) Incubation of B-CLL cells with CD154+/gp350+ exosomes for two days led to the induction of immune accessory molecules CD54, CD80, CD86 and CD95. One representative experiment of at least ten is shown.

In a next series of experiments we wanted to elucidate whether 293/gp350+ exosomes can co-transfer functional membrane proteins to B cells. As a model system but also as a potential practical application, we chose B-CLL cells that express CD21 and become immunogenic upon ectopic expression of CD154 on malignant cells. We, therefore, transfected 293 cells with expression plasmids for gp350 and CD154 and isolated exosomes as described above. Again, an immunoblot with a CD154-specific antibody demonstrated the presence of CD154 in exosome preparations and an OptiPrep™ gradient revealed co-sedimentation with gp350 (Figure 1A) indicating the presence of both proteins on exosomes (Figure 2A). In addition, flow cytometry revealed that gp350+/CD154+ exosomes specifically bound to CD19+ B cells from a B-CLL patient (Figure 2B).

To answer the question whether CD154 in gp350+/CD154+ exosomes was functional, HEK293 cells were transfected with a CD154 expression plasmid either alone or in combination with a gp350 expression plasmid. Transfer of exosomes to B-CLL cells was measured with an anti-CD154 antibody by flow cytometry. As shown in Figure 2C, exosomes that carry both proteins efficiently conveyed CD154 surface expression to B-CLL cells. CD154+/gp350– exosomes were only transferred very inefficiently to B-CLL cells, probably due to an only weak interaction of CD154 with its receptor CD40 on the cell surface. In order to investigate the immune accessory function of CD154, we loaded B-CLL cells from an HLA II DR13+ donor either with 293 exosomes, with exosomes from 293 cells transfected with an expression plasmid for gp350 (gp350+ exo) or transfected with expression plasmids for gp350 and CD154 (CD154+/gp350+ exo). One day later, we added HLA-DR13-restricted gp350-specific CD4+ T cells and measured their activation with an IFN-γ ELISA. This assay demonstrated the relevance of CD154 for T-cell recognition because B-CLL cells loaded with CD154+/gp350+ exosomes were significantly better stimulators than B-CLL cells loaded with gp350+ exosomes (Figure 2D). Induction of the accessory molecule ICAM-1 (CD54), the co-stimulatory molecules B7.1 (CD80) and B7.2 (CD86), and the death receptor Apo1 (CD95) on B-CLL cells upon incubation with CD154+/gp350+ exosomes (Figure 2E) but not with exosomes from 293 cells provided an explanation for improved recognition by T lymphocytes.

### gp350 is a neo-antigen in B-CLL cells and activates specific CD4+ cytolytic T lymphocytes

**Figure 3:**
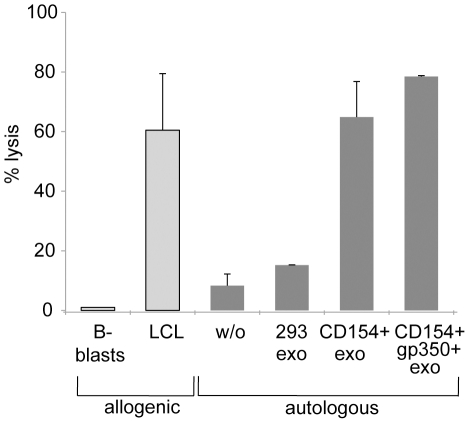
CD154+/gp350+ exosomes reactivate gp350-specific T cells. PBMCs from a patient diagnosed with B-CLL were stimulated three times with CD154+/gp350+ exosomes and then used as effector cells towards autologous calcein-labeled PBMCs loaded with the exosomes indicated or not loaded with exosomes (w/o). PBMCs loaded with CD154+/gp350+ exosomes were constantly better targets than PBMCs loaded with CD154+ exosomes. Stimulated PBMCs were also used as effector cells towards allogenic EBV+ LCL and EBV– B-blasts. The fact that the LCL was efficiently lysed by stimulated PBMCs whereas the B-blasts were not further indicates the reactivation of gp350-specific T-cells. One out of three independent experiments are shown.

We knew from previous experiments that PBMCs from healthy donors, which were incubated with gp350+ exosomes, efficiently re-stimulated an autologous gp350-specific CD4+ T-cell clone (data not shown). These experiments implicated that gp350, which is equally transferred to B-CLL cells by CD154+/gp350+ exosomes, can act as a neo-antigen in B-CLL cells, which are normally not infected with EBV [18]. This is a very interesting aspect because B-CLL patients usually maintain a robust and easily recruited CMV- [19] and EBV-specific T-cell response [2] although their T cells in general are functionally impaired. For instance, B-CLL cells infected with EBV or loaded with CMV-peptides are efficiently killed by autologous T lymphocytes from B-CLL patients [18], [20]. We thus aimed at investigating whether gp350 serves as a viral neo-antigen in exosome-treated leukemic cells and whether these cells become targets for gp350-specific autologous T lymphocytes. We stimulated PBMCs from B-CLL patients three times with CD154+/gp350+ exosomes as described above and elucidated the specificities and cytolytic potential of the activated T cells. To this end, we incubated autologous PBMCs for 24 hours with either CD154+/gp350+ exosomes, CD154+/gp350– exosomes or unmodified 293 exosomes or left them untreated. The next day, these cells were labeled with calcein and used as targets for autologous effector T cells. As shown in Figure 3, T cells generated by stimulation with gp350+/CD154+ exosomes efficiently killed autologous PBMCs that had been activated with CD154+/gp350– or CD154+/gp350+ exosomes as quantified by the release of calcein. Of interest, we did not observe notable activation of T cells against 293 proteins as PBMCs incubated with exosomes from non-transfected 293 cells were lysed to almost the same extent as non-activated autologous PBMCs.

These experiments also demonstrated that PBMCs loaded with CD154+/gp350+ exosomes were constantly better lysed than PBMCs loaded with CD154+/gp350– exosomes. This prompted us to investigate whether T cells specific for gp350 accounted for this improved lysis. Therefore, we tested the cytolytic activity of these T cells against EBV-infected lymphoblastoid cells (LCLs), which are known to be targets for gp350-specific T cells [21], and against EBV-negative B blasts [22] from an HLA-DR13+ matched healthy donor. We found that the T cells efficiently lysed allogenic LCLs, whereas they completely ignored EBV-free B blasts, indicating the presence of EBV-specific T cells in the effector population (Figure 3). These data corroborate that the stimulation of PBMCs with CD154+/gp350+ carrying exosomes is an efficient method for the activation of B-CLL cells and the reactivation and expansion of CLL- and EBV-specific T lymphocytes in B-CLL patients.

### B-CLL cells treated with CD154+/gp350+ exosomes are potent stimulators of tumor-specific autologous T cells

**Figure 4:**
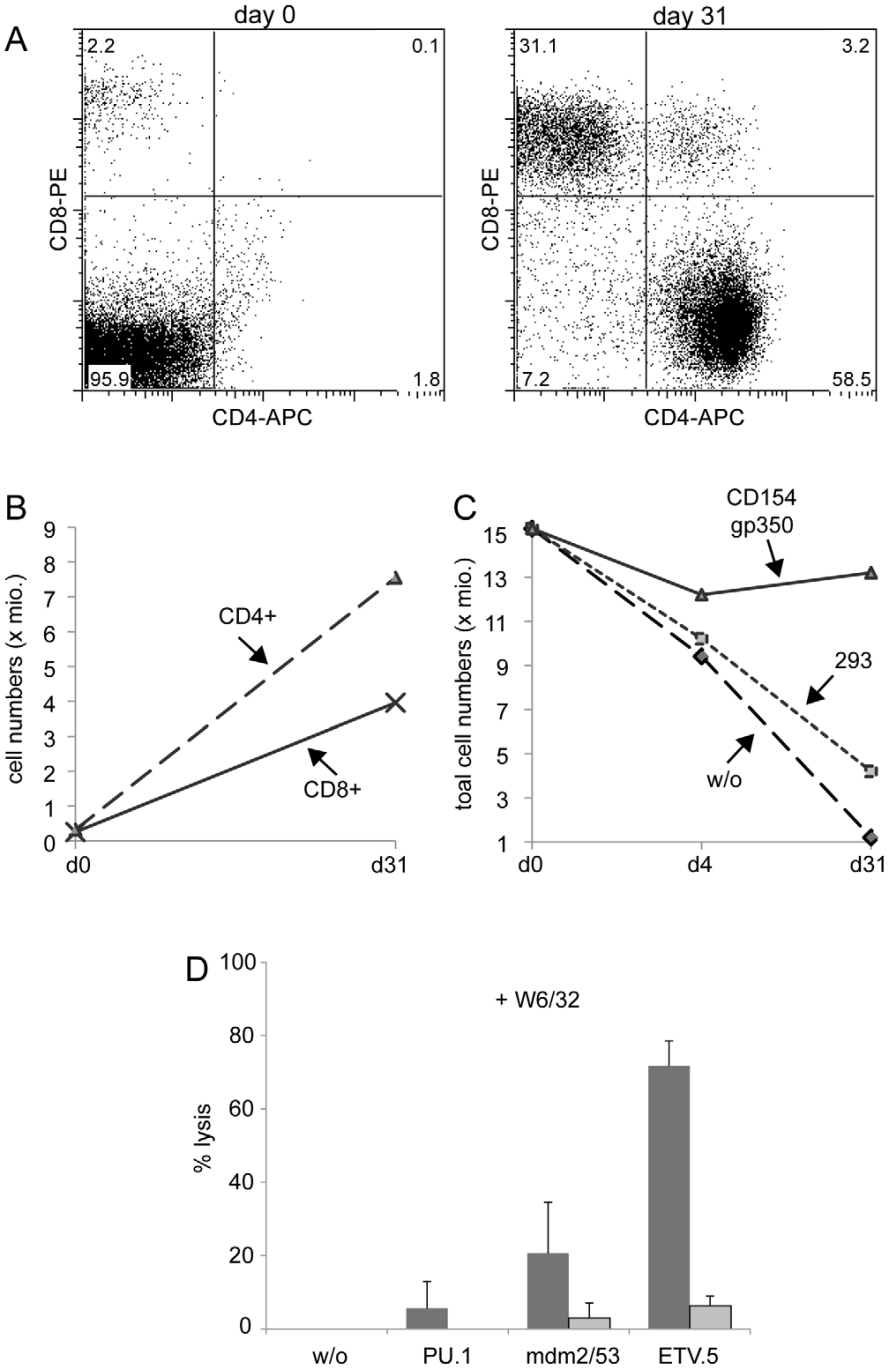
Stimulation with CD154+/gp350+ exosomes reactivates autologous T-cells from patients with B-CLL. Peripheral blood from a patient with only 4% T-cell among his leukocytes at day 0 (left) were stimulated three times with different exosomes within a period of 31 days, yielding a cell population that consisted to 58% of CD4+ and 31% of CD8+ T-cells (B). The stimulation of PBMCs with CD154+/gp350+ exosomes led to a 23-fold and 15-fold increase in numbers of CD4+ and CD8+ T-cells, respectively, while (C) the total cell number slightly declined. No vital cells were detectable after 31 days when PBMCs were stimulated with exosomes from parental 293 cells (293) or when cells were left untreated (w/o). One representative experiment of five is shown. (D) Stimulation of B-CLL with CD154+/gp350+ exosomes let to the stimulation of tumor-specific T cells that lysed allogenic, calcein-labeled B-blasts, loaded with A2-restricted peptides derived from known CLL-associated antigens (black columns) but not unloaded B-blasts (grey columns). Peptides were derived from PU.1, mdm2 or ETV.5, which were all described as CLL-associated antigens. Blocking of lysis upon addition of the MHC class I-blocking antibody W6/32 indicates MHC class I specificity and defines CD8+ T-cells as major effector cells. One representative experiment of three is shown.

Having demonstrated that B-CLL cells activated by CD154+/gp350+ exosomes achieve an activated phenotype, we next wanted to assess whether these B-CLL cells became immunogenic to autologous EBV- and CLL-specific T cells. Given the expected low number of these T cells in peripheral blood, we stimulated 1,5×10^7^ PBMCs from patients with B-CLL three times within 21 days with lethally irradiated autologous PBMCs that were incubated with different exosomes as described above. One typical experiment is shown in Figure 4: before the first stimulation on day 0, PBMCs of this patient consisted mainly of malignant B cells with only 4% CD3+ T cells. After the third round of stimulation, cultures incubated with gp350+/CD154+ exosomes almost exclusively contained CD4+ and CD8+ T-cells (Figure 4A). In total, stimulation with CD154+/gp350+ exosomes yielded approximately 1×10^7^ vital cells on day 31, meaning that the CD4+ and CD8+ T cell counts had increased about 25-fold and 15-fold, respectively (Figure 4B), while the total cell number slightly decreased (Figure 4C). In contrast, no viable cells were detectable in those cultures that were treated with CD154–/gp350– exosomes derived from non-transfected 293 cells, or that were left untreated. Thus, stimulation of T cells with B-CLL cells loaded with CD154+/gp350+ exosomes is a powe rful option to selectively expand specific T cells from B-CLL patients *in vitro.*

Next we wanted to elucidate the specificities and cytolytic activity of these T cells in more detail. As mentioned above, re-stimulated T cells form B-CLL patients efficiently lysed autologous PBMCs that were activated with CD154+/gp350– exosomes. These exosomes do not contain gp350 and, therefore, the T cells must have recognized cellular antigens. To test whether T cells specific for B-CLL-associated antigens had been reactivated, we incubated them with irradiated EBV-negative B-blasts [22] from an HLA-A2-matched donor as a negative control or B-blasts loaded with HLA-A2 restricted peptides derived from B-CLL-associated antigens, namely MDM [23], ETV5 [24] and PU.1 [25]. As shown in Figure 4D, peptide-loaded B-blasts were efficiently lysed by the T cells stimulated with CD154+/gp350+ exosomes whereas B blasts not loaded with peptides were completely ignored. Lysis of target cells was efficiently reduced upon addition of an MHC class I-specific antibody, W6/32, demonstrating the antigen specificity of the effector T cells.

## Discussion

CD154 is a known promising candidate molecule for the immunotherapy of B-CLL because it increases the immunogenicity of the tumor cells. In line, the ectopic expression of CD154 on B-CLL cells was shown to induce the expression of important co-stimulatory and adhesion molecules on the leukemic cells, turning them into efficient stimulators of autologous T cells. In principle, viral gene transfer of CD154 into B-CLL cells is a suitable approach to induce immunological reactions both *in vitro* and *in vivo* [14], [26]–[29] but the low transduction efficiency and the resulting high dose of viral vectors may induce severe side effects. There also remain major concerns about the clinical use of viral vectors.

Exosomes are cellular microvesicles, which have already demonstrated their potential to induce specific immune responses. The majority of permanent cell lines spontaneously release exosomes into the supernatant, from where they can be easily purified and concentrated [30]. Transfecting HEK293 cells with expression plasmids coding for CD154 and gp350 resulted in the secretion of modified exosomes carrying both proteins. Upon interaction with their target B cells, an undetermined fraction of exosomes is presumably engulfed and degraded in lysosomes in such way that peptides of exosomal proteins are presented in association with MHC class II molecules. Exosomes that are not taken up by the target cells probably engage in protein-protein contacts with cellular surface molecules and lead to receptor activation and signaling. gp350 molecules on the surface of exosomes probably target these vesicles exclusively to human B cells which express relevant amounts of the receptor molecule for gp350, CD21. Exosomal co-transfer of both gp350 and CD154 is thought to lead to efficient CD154/CD40 ligand/receptor engagement on the B-cell surface activating the intrinsic CD40 signaling cascade.

Treatment of B-CLL cells with exosomes transferring functional CD154 protein causes leukemia cells to become efficient APCs. Activating the CD40 receptor leads to the induction of immune accessory molecules, making these leukemic cells potent stimulators of autologous T lymphocytes. Since almost all patients with CLL have a high prevalence of EBV (94%) [31] and thus possess EBV-specific memory cells with a high frequency of CD4+ gp350-specific T cells [32], the incorporation of gp350 into exosomes has a dual function: it confers B-cell tropism and serves as an immunodominant viral CD4 antigen. The incorporation of a viral protein as a tumor-specific antigen is straightforward, because the cellular immune system of cancer patients is usually impaired but virus-specific immune responses are detectable even in late-stage patients and T lymphocytes specific for herpes viruses are often present at high numbers [1], [33]. Given the fact that the vast majority of CLL patients are seropositive for EBV, gp350 and other EBV proteins have the potential as promising neo-antigens in B-CLL cells exploiting and redirecting the strong anti-viral cellular immune responses to leukemic cells.

Based on these results, we propose here a new immunotherapeutic approach for B-CLL, based on the simultaneous targeted transfer of functional CD154 and the EBV protein gp350 onto malignant cells using exosomes. gp350 is the major envelope protein of EBV and confers viral B-cell tropism by interacting with the complement receptor 2 (CD21), which is highly expressed on B lymphocytes. Here, we generated modified exosomes produced in 293 cells. As a result of gp350 incorporation, these particles have a profound B-cell tropism similar to wild-type EBV, so that gp350-carrying vesicles specifically and efficiently bind to B cells. In addition, gp350 serves as a viral neo-antigen in B-CLL cells. We also found that CD154 on exosomes from HEK293 cells is functionally active as demonstrated by the induction of immune accessory molecules on B target cells probably through the CD40 pathway. Taken together, our experiments suggest that leukemia cells treated with CD154+/gp350+ exosomes are efficiently stimulated and subsequently killed by autologous B-CLL and gp350-specific cytolytic T lymphocytes.

In summary, our results demonstrate that modified exosomes carrying the EBV protein gp350 display a distinct tropism to normal and leukemic B cells and efficiently transfer CD154 as a functional protein onto these cells. Leukemic B cells treated with these particles acquire an activated phenotype and become potent stimulators of autologous T lymphocytes. Engineered exosomes can be easily generated and can readily be scaled up for clinical applications. In addition, they can be individually tailored to express additional accessory molecules like OX40L or the Fas ligand or alternative viral molecules to target other classes of cells like macrophages and DCs. The generation of modified exosomes is not limited to 293 cells, which we used in this proof-of-concept. Instead, other cell lines that are approved for human therapy, such as MRC-5 fibroblasts, should also be tested as an optional origin of exosomes to facilitate transition into clinical trials. Modified gp350-carrying exosomes can thus be regarded as powerful and promising tools for various immunotherapeutic approaches.

## Materials and Methods

### Blood samples and cell preparation

Peripheral blood samples were obtained from patients with diagnosis of B-CLL after informed consent approved by the Institutional Ethics Committee. Normal blood samples were taken from healthy volunteers. Mononuclear cells were isolated by density gradient centrifugation on F/H. B cells were purified with CD19-specific MACS beads (Miltenyi, Bergisch Gladbach, Germany). Cells were cultured at 37°C in a 5% CO_2_ atmosphere in standard medium with 10% fetal calf serum.

### Isolation of exosomes

HEK293 is a human embryonic kidney cell line [34], which spontaneously releases exosomes into the cell culture supernatant. For the generation of modified exosomes, 293 cells were co-transfected with expression plasmids for BLLF1 (gp350), CD154 and/or gfp. For the isolation of exosomes, 25 ml of conditioned supernatants were collected three days later, sterile filtrated and subjected to repeated centrifugations at increasing centrifugal force (10 min at 300 x g, 10 min at 5000 x g in a Heraeus 3SR+-centrifuge, followed by 2 h at 100.000 x g in a Beckman LE-80K ultracentrifuge in a SW28 swing-out rotor. The pelleted particles were washed, resuspended in 500 µl volume PBS containing protease inhibitors (Complete Mini, Roche) and the protein content was analyzed in a Lowry microassay using reagents purchased from Bio-Rad (Munich, Germany). Exosomes were further purified by flotation into a 5-30% iodixanol gradient (OptiPrep™, Sigma Aldrich, Deisenhofen, Germany).

### Binding assays and flow cytometry

B-CLL cells were cultivated with 100 µg of exosomes in a final volume of 2 ml for two days. Induction of surface accessory molecules was measured by flow cytometry using a FACS Calibur flow cytometer (Becton Dickinson). For bead-coupling assays 5 µl of surfactant-free sulfate/aldehyde latex beads were incubated with 10 µg of exosomes for 15 min at room temperature. Then 1 ml of PBS was added and the beads were incubated for another 2 hours. The beads were washed three times in PBS with 2% FCS and analyzed. Antibodies specific for tsg101 (sc-978) and CD154 (sc-7964) were purchased from Santa Cruz Biotechnology. E. Kremmer (Munich) provided the gp350-specific antibody 72A1. Fluorochrome-labeled secondary antibodies were obtained from Becton Dickinson (Heidelberg, Germany) or Immunotools (Friesoyte, Germany).

### Immunoblots

Vesicle preparations were spotted onto a PVDF membrane, incubated with with specific primary antibodies and an HRP-coupled secondary antibody and developed with the ECL system (GE Healthcare). Ganglioside M1 was detected with colera toxin (Sigma Aldrich).

### T cell reactivation assays

In order to reactivate EBV-specific T-cells in B-CLL blood samples, 3×10^7^ cells were cultivated with 100 µg of exosomes in a final volume of 5 ml. Re-stimulations were performed on days 14 and 28 by adding 1×10^7^ lethally irradiated autologous PBMCs that have been loaded with 100 µg exosomes for 5h. Fresh medium was added once a week. After 31 days, cells were analyzed by flow cytometry for lineage markers. Interferon-γELISA assays were performed according to the manufacturers instructions (Mabtech, Uppsala, Sweden). The gp350- and BNRF1-specific CD4+ HLA-DR13-restricted T-cell clones used have been described elsewhere [8], [35]. All cells were tested in triplicates.

### Cytotoxicity assays

Calcein release assays were performed as described previously [36]. Briefly, target cells were labeled with Calcein-AM (1% solution in medium) for 30 min at 37°C. After intensive washing the cells were either incubated with 10 µg of exosomes for 4 h or loaded with CLL-specific, HLA-A2-restricted peptides (PU1.423: aa-sequence VLFYLGQYI, mdm2.53: YLAPENGYL and ETV5.45: ELFQDLSQL) at 20 µg/ml for 1 h or left untreated. The cytotoxicity assays were performed over a time period of 6 h, using an effector-target-ratio of 40∶1, and the calcein released into the supernatant was quantified with excitation and emission wavelengths of 490 nm and 530 nm, respectively, in a plate-reader (Perkin Elmer, Waltham). All experiments were performed at least three times.
